# The influence of cognitive control on the processing of L2 garden path sentence among Chinese–English bilinguals

**DOI:** 10.3389/fnbeh.2022.976155

**Published:** 2022-09-23

**Authors:** Zhilong Xie, Guofang Zeng, Shuya Zhou, Juan Wang

**Affiliations:** Foreign Languages College, Jiangxi Normal University, Nanchang, China

**Keywords:** cognitive control, working memory, mental set shifting, conflict monitoring, inhibition, garden path sentence comprehension

## Abstract

Few studies have examined the role of cognitive control in processing ambiguity, let alone the roles of different components of cognitive control. In the current study, the English (L2) Sentence Processing Task and a series of cognitive control tasks were administered among 111 young adult Chinese–English bilinguals to investigate the influence of different components of cognitive control on garden path sentence comprehension, with other factors such as age, socio-economic status, and language proficiency strictly matched. Data analysis results showed a significant garden path effect on response times (RTs) and accuracy among all the participants. The results of independent *t*-test analyses revealed that the high working memory (WM) group was faster in ambiguity resolution, and so was the high monitoring group. However, there were no differences between the high and low inhibition and shifting groups in ambiguity resolution. These findings reveal that only certain aspects of cognitive control influence garden path sentence comprehension.

## Introduction

Sentence comprehension is believed to be an incremental process (Demberg and Keller, 2019), during which readers constantly construct temporary and changeable meaning before they arrive at the end of the sentence. Garden path sentence, however, also known as one type of syntactically ambiguous sentence (Lee, 2006), would lead readers along different paths before they form accurate comprehension. Chances of falling into the tricky trap of such ambiguous sentences are great for readers in most cases. One of the most distinctive features of such sentences, as suggested by many scholars, is something luring readers into a very probable interpretation, which later turns out to be incompatible with the end of the sentence (Besserman and Kaiser, 2017; Masia et al., 2017; Yoo et al., 2017; Demberg and Keller, 2019). The garden path sentence has received great attention in the literature. Concerning the potential factors contributing to the comprehension of garden path sentences, most studies focused on individuals’ language proficiency, including basic words, syntactic knowledge, and thematic roles (e.g., Ferreira and Henderson, 1991; Christianson et al., 2001; Brothers et al., 2021). However, few studies have examined the role of general cognitive control in ambiguous sentence processing, i.e., garden path sentence comprehension (e.g., Choi and Trueswell, 2010; Hussey et al., 2017).

Cognitive control is an essential mental cognitive process that human beings need to enable appropriate goal-directed behavior through the regulation of basic thoughts and actions. Cognitive control, as a complex construct, has been suggested to include multiple components (Miyake et al., 2000; Hussey et al., 2017), which can be classified into dimensions such as inhibition, mental set shifting, updating, monitoring of working memory, and conflict monitoring (Miyake et al., 2000; Lee, 2006; Green and Abutalebi, 2013). In the last two decades, a small number of studies have focused on the relationship between cognitive control and the comprehension of garden path sentences (e.g., Choi and Trueswell, 2010; Key-DeLyria and Altmann, 2016; Pozzan and Trueswell, 2016; Yoo and Dickey, 2017). Particularly, most of such studies investigated the role of working memory, a subcomponent of cognitive control, in processing ambiguous sentences (e.g., Teubner-Rhodes et al., 2016; Hussey et al., 2017; Yoo and Dickey, 2017). Few have ever examined the roles of other components of cognitive control in garden path sentence comprehension. With this understanding, the current study intends to fill the gap by exploring the roles of different components of cognitive control in garden path sentence comprehension, i.e., the roles of working memory, inhibition, conflict monitoring, and mental set shifting.

### Garden path sentence

The phenomenon of the garden path effect is firstly noticed by psycholinguist Bever (1970), who defines such syntactically ambiguous sentences as garden path sentences. The occurrence of the garden path effect phenomenon arises when parsers, scanning from left to right, form one possible interpretation of the sentence, but find it to be mismatched with the correct meaning, and then go back to resolve the ambiguity by adopting another interpretation, thus resulting in difficulty and longer processing time for the rest of the sentence (Carroll, 2000). Therefore, a reanalysis becomes extremely necessary to achieve the correct interpretation. For example:

*(1) Without her contributions failed to appear.*

While scanning this sentence, the parser would prefer to classify “contribution” to “her” as the object of the preposition “without.” Consequently, the verb “failed” may have no subject, resulting in processing failure. The parser may return from the beginning to analyze the sentence again. In such sentence processing, readers are looking for the right path in a garden distracted by lures and returning for another path. However, people prefer to comprehend ambiguous sentences in a most acceptable other than a devious way, which is nothing but the correct interpretation. In essence, it is true that the garden path sentence, indeed, is a kind of ambiguous sentence, which is featured in syntactic ambiguity. Therefore, it could be directly regarded as a synonym for syntactically ambiguous sentences, but not all syntactic ambiguity will lead to processing failure (Lee, 2006). For example:

*(2) The boy shot the man with the gun.*

Syntactic ambiguity would be found in this sentence, which could be interpreted as “The boy shot a man equipped with the gun” or “The boy shot the man by gun.” Those two interpretations are both acceptable, so there is no need for reanalysis. In the contrast, there is only one correct understanding/interpretation of the garden path sentence. There are three types of garden path sentences, which are main verb/reduced relative clause (MV/RR), direct object/subject ambiguity (DO/S), and direct object/sentence complement ambiguity (DO/SC). Corresponding examples could be seen below:

*(3) The horse raced past the barn fell. (MV/RR)*

*(4) While the man hunted the deer ran into the woods. (DO/S)*

*(5) Jane convinced her parents are interested in their children. (DO/SC)*

In sentence (3), the parser would mistakenly group the prepositional phrase “raced past the barn” with the subject “the horse” as an “NP + V + PP” structure before getting confused about the extra verb “fell” at the end of the sentence, whereas in sentence (4), garden path effect would arise when the parser classifies “the deer” as the direct object of “the man hunted,” other than the subject of “ran into the woods”. Similarly, in sentence (5), the chances of taking “her parents” as the direct object of the verb “convinced” are so strong before the parser later encounters another verb “are” and realizes the so-called direct object should be the subject of the predictive clause [i.e., Jane convinced her (that) parents are interested in their children]. In short, based on the examples above, the garden path sentence must follow three conditions: (1) the initial interpretation of the front part of the sentence may lead to the processing failure of the rest part of the sentence; (2) reanalysis is consciously or unconsciously needed; (3) there is only one correct interpretation.

There are many studies in the literature aiming at investigating the garden path sentence (e.g., Ferreira and Henderson, 1991; Juffs and Harrington, 1996; Waters and Caplan, 1996; Ferreira et al., 2001; Christianson et al., 2006; Fujita, 2021; Huang and Ferreira, 2021). The garden path effect is prevalent in both native and non-native languages, i.e., English for native speakers and German speakers (Jacob and Felser, 2016). Pozzan and Trueswell (2016), similarly, examined whether adult L2 learners experience the garden path to a similar degree as native adults. They found that L2 speakers of English experienced increased difficulties revising initial interpretations, which is similar to those observed for 5-year-old native children. It is proposed that L2 learners’ difficulties with revision may be related to increased recruitment of cognitive control for L2 learners who do not have a high proficiency. Moreover, in a non-syllabic language such as Chinese, the garden path effect is also observed, although studies in this area are few (e.g., Jin, 2006; Hsieh et al., 2009; Hsieh and Boland, 2015). For example, Hsieh and Boland (2015) examined whether readers maintained multiple interpretations throughout the ambiguous region or selected a single interpretation at the point of ambiguity. The results revealed that the garden path effect could be modulated by the strength of support (i.e., RC-relative clause vs. CC-complement clause), and the processing difficulty was greater for the case when the RC interpretation was much more strongly supported.

However, why and how the garden path effect happens remains open. Besides the incremental steps of language processing in garden path sentences (as discussed above), other studies have shown that the comprehension of garden path sentences could be attributed to many factors, such as language proficiency and demographics (e.g., age, IQ) (e.g., Besserman and Kaiser, 2017; Engelhardt et al., 2017; Yoo and Dickey, 2017). Language proficiency, for example, is treated as one of the most significant factors leading to accurate interpretation, even among L2 learners (Cheng, 1998; Gu and Cheng, 2010; Zeng, 2016). Individuals with lower L2 proficiency, who possess a relatively smaller range of vocabularies, may have more difficulties in garden path sentence comprehension. More recently, however, psycholinguists have attempted to explore what happens in cognition when processing garden path sentences and found that accurate and precise sentence processing is attributed not only to language-related factors but also to the cognitive control ability of an individual (e.g., Ye and Zhou, 2008; Novick et al., 2014), which suggests the necessity of further research regarding the relationship between cognitive control mechanism and the comprehension of garden path sentence, beyond language itself.

### Cognitive control and garden path sentence processing

Multiple studies have revealed that general cognitive control is recruited during language comprehension both at the behavioral level and at the neural level, but many aspects of this relationship remain elusive (e.g., Novick et al., 2009; Vuong and Martin, 2011; Fedorenko, 2014). Previous studies have largely focused on the relationship between working memory and garden path sentence comprehension. Relatively speaking, among the components of cognitive control, the majority of studies focused on working memory, which was measured usually by reading span task (Engelhardt et al., 2017). Early in 1996, Waters and Caplan compared groups with different verbal working memory spans but failed to find differences in their performance on garden path sentence comprehension. Later on, quite a few empirical studies reported a positive correlation between working memory and garden path sentence comprehension (e.g., MacDonald et al., 1992; Christianson et al., 2006; Gu and Cheng, 2010; Teubner-Rhodes et al., 2016; Hussey et al., 2017; Yoo and Dickey, 2017). For example, MacDonald et al. (1992) proposed that the working memory capacity of a reader constraints/affects the processing of lexical or syntactic ambiguity, which is later supported by empirical studies (e.g., Miyake et al., 1994; Gadsby et al., 2008). Furthermore, some recent studies have explored the causal role of cognitive control in garden path sentence processing (e.g., Hussey et al., 2017; Hsu et al., 2020). In one such study by Hussey et al. (2017), the authors designed a complete working memory training system by using different versions of the N-back task to observe participants’ performance on garden path sentences in pretest and post-test. Fifty-nine balanced bilinguals and 51 monolinguals were randomly assigned to two versions of the 3-back task (high-conflict: 32 bilinguals and 26 monolinguals; low-conflict: 27 bilinguals and 25 monolinguals) before taking 20-min training. The results showed that high-conflict training—but not low-conflict training—brought about benefits on several untrained transfer tasks, but only under selective conditions requiring cognitive control, which suggests that domain-general cognitive control mechanisms may play a causal role in linguistic and non-linguistic performance. This result is significant in that it clarifies the cause and effect interplay between cognitive control and ambiguity sentence processing, whereas most previous studies are correlational.

Investigations of the roles of other components of cognitive control in garden path sentence processing are very few. Only several relevant studies can be referred to. For example, shifting is believed to participate in the reanalysis and reconstruction of the sentence comprehension process and help the individual decide between various potential interpretations (Key-DeLyria and Altmann, 2016). Woodard et al. (2016) investigated how individual differences in children’s ability to interpret temporarily ambiguous sentences relate to individual differences in other linguistic and domain-general cognitive abilities. The children participants completed a series of tests measuring working memory, cognitive flexibility, and language comprehension. The results showed that two measures of cognitive flexibility were related to their ambiguity resolution abilities, i.e., switching in the Card Sorting test and switching cost in the Flanker, which suggests that shifting ability has a significant role in ambiguity resolution. Conflict monitoring is also believed to play a crucial role in garden path recovery (Teubner-Rhodes et al., 2016). According to Botvinick et al. (2001), the detection of conflict automatically triggers cognitive control mechanisms, which can enhance the resolution of subsequent conflict in sentences containing ambiguities or conflicts. Cognitive control may play a role by monitoring when processing has gone awry and then initiating behavioral adjustments accordingly (Hsu and Novick, 2016). In addition, the component of inhibiting ability is also reported to be closely related to sentence processing (Eriksen and Eriksen, 1974; May et al., 1999). According to Vosse and Kempen (2000), to comprehend language (particularly ambiguous utterances), the parser operates in conjunction with lexicalist grammar and competitive inhibition, in which the target words should be activated but the non-target irrelevant words should be suppressed. However, empirical studies concerning garden path sentence comprehension are limited. Vuong (2008) revealed the involvement of inhibitory control in the recovery of garden path sentences in two self-paced reading experiments. Other studies may reveal an indirect relation between inhibition and garden path sentence processing. For example, Yoo and Dickey (2017) conducted an experiment by measuring the working memory (by sentence-span, forward- and backward-digit span tasks (DST) as well as subtract 2-span task), and inhibiting ability (by Flanker task). The results revealed that older adults, who had a disadvantage in cognitive control tasks measuring working memory and inhibition, were significantly slower than younger adults on online garden path sentence processing, and working memory but not inhibitory control predicted off-line processing. However, the literature on the relationship between inhibition in cognitive control and garden path sentence comprehension is rather rare.

Overall, when cognitive control is treated as a unitary construct in the processing of a garden path sentence, the immature or deficit cognitive control ability of readers could get in the way of revising after forming an initial misunderstanding of the ambiguous sentence. The study of Choi and Trueswell (2010) compared 16 developing children and 16 adults and found that the children, whose cognitive control ability was immature, were worse in garden path sentence processing. Worse garden path processing was also found among adults whose cognitive control resources were limited when L2 proficiency was not fully proficient. In Pozzan and Trueswell (2016), 33 adult Italian–English bilinguals and 30 adult monolinguals participated in the eye movement experiment. The results revealed that L2 learners’ act-out patterns were overall less accurate than those of native speakers, and their performance was with high error rates, particularly in response to temporarily ambiguous sentences, which was thought to stem from L2 learners’ cognitive depletion/overload of the cognitive control network as a result of competing for the same set of cognitive resources for a not fully proficient language. Therefore, it is safe to believe when dealing with conflict-resolution tasks like garden path sentence comprehension, the involvement of cognitive control is extremely necessary. A better cognitive control seems to facilitate the garden path sentence processing.

To sum up, when investigating the processing of the garden path sentences, it is highly necessary to verify the role of cognitive control. Recent neuro-image studies suggest that during sentence processing, some cognitive control-related brain areas are also activated, such as the left dorsolateral prefrontal cortex (DLPFC) (Klaus and Schutter, 2018). Cognitive control would help readers realize the potential decision before taking the right path toward the accurate meaning of ambiguous sentences (Novick et al., 2014). However, as reviewed above, most previous studies focused on the relationship between working memory and garden path sentence processing but did not take multiple measuring tasks to examine how different components of cognitive control are related to the processing of garden path sentences, particularly among L2 learners. Therefore, the current study intends to fill the gap by exploring whether different levels of cognitive control would lead to different performances of garden path sentence processing. In particular, two questions are of our concern:

RQ1: Do Chinese EFL students encounter the so-called “garden path effect”?

RQ2: If so, do different components of cognitive control (i.e., mental set shifting, working memory, conflict monitoring, and inhibition) differentially affect garden path sentence processing?

## Materials and methods

### Participants

Altogether 111 healthy L2 (English) learners from Jiangxi Normal University in China participated in the current study. As English is considered a foreign language in China, our participants learned the language mostly in class and were typical foreign language learners who did not speak it for communication purposes on daily basis. All the participants were between 21 and 28 years of age (*M* = 21.33, *SD* = 2.15), and were from first year to fourth year students (*N*_1_ = 35; *N*_2_ = 28; *N*_3_ = 19; *N*_4_ = 29), among which 10 were male students and 101 were female students. All participants had passed the college entrance examination, some of whom even passed TEM-4 (Test for English Majors-Band 4) or TEM-8 (Test for English Majors-Band 8) and had been learning English as a second/foreign language for more than 10 years. Therefore, they had acquired a basic understanding of English semantic and syntactic knowledge and could comprehend ambiguous structures like garden path sentences in this research. The participants voluntarily took part in this study for course credit with written informed consent, and their rights were well protected according to the ethics approved by the Academic Committee of the university.

### Demographic measures

#### Socio-economic status

Evidence has shown that cognitive control is modulated by demographic factors like age, socio-economic status (SES), etc. (Xie and Zhou, 2020). Therefore, those demographic features of our participants were also collected. In particular, since our participants were still college students without stable jobs and income, their paternal, as well as maternal education was treated as an approximate indicator of SES on a scale from 1 to 7, with 1 indicating nearly illiterate and 7 indicating doctoral degree.

### Language proficiency test

In this research, a questionnaire on language learning background was administered (Marian et al., 2007), in which both first and second language proficiency of participants was measured by a self-rated 10-point Likert scale. The highest score is 10 while the lowest is 1 (i.e., “10” conveys being perfect in this skill while “1” conveys having little knowledge of that skill). This self-rated measurement is widely used in bilingual research and is reported to have high correlations with objective language proficiency (Prior and Gollan, 2011). Apart from the self-rated scale, an L2 Semantic Verbal Fluency Test was adopted to measure the objective L2 proficiency, during which participants were required to produce as many words as possible in each category (jobs, animals, furniture) within 60 s. The test has been reported to be a good indicator of vocabulary size (Bialystok et al., 2009). L1 was not objectively measured since our participants were native L1 speakers, although variations might exist.

### Sentence processing task

The materials used in the sentence processing task included L2 (English) garden path sentences and filler sentences. The garden path sentence types were main verb/reduced relative clause (MV/RR), direct object/subject ambiguity (DO/S), and direct object/sentence complement ambiguity (DO/SC). Altogether there were 32 sentences in the task, of which 16 were garden path sentences (e.g., The horse raced past the barn fell) and 16 were normal sentences (e.g., The boy named Tom is my brother). The two types of sentences were presented in the second language (English) and were of similar length and vocabulary difficulty. They were presented in a mixed order so that participants were put in a random situation where cognitive control was highly needed to form a correct judgment whenever confronted with a garden path sentence. Each sentence was followed by a comprehension question to check whether the participant had fully understood the target sentence.

The entire experiment was composed of practice and experimental blocks. Each trial started with a fixation of “ + “ for 1000 ms. A randomly presented sentence would appear for 5000 ms or until the designed key was pressed by the participant. Then a question was presented for 5000 ms to check whether the comprehension of the garden path sentence was correct or not. Participants were required to judge true or false by pressing the designated key, according to the sentence presented earlier. This task was designed via E-Prime 2.0. Reaction times and accuracy were recorded by the computer.

### Cognitive control tasks

#### The digit span task

As one of the simple memory span tasks, the DST is generally composed of two modalities, which are Digits Forward and Digits Backward. Evidence from neuropsychological research and clinical evaluations suggests DST not only helps identify certain brain damage areas of the patients, like left cerebral lesions (Heilbronner et al., 1991), but also serves as the measure for immediate verbal recall and attentional capacity (Ostrosky-Solís and Lozano, 2006). In this research, participants were only required to complete the Digits Forward task for working memory capacity. The Visual Digits Forward task, which was adopted from http://www.millisecond.com (based on Woods et al., 2011), consisted of the practice part and the formal experimental part. Participants were required to correctly complete the practice part, and then they could be allowed to enter the formal experimental part, which included 14 trials. Each trial started with a fixation “+” for 1000 ms, with each digital number appearing for 1000 ms on the computer screen. Participants were required to select those digits in the correct order by clicking the mouse. After clicking and submitting, it automatically moved on to the next trial. This test started with three digits in the beginning. If three digits level was achieved with above 50% accuracy, then it moved up to a higher level (i.e., 4, 5, 6). This task was designed and operated on Inquisit Lab 6.

#### The Wisconsin card sorting test

The Wisconsin card sorting test (WCST) (Heaton, 1981) was designed for measuring mental set shifting of cognitive control, which required participants to match a response card to one of four stimulus cards based on different attributes (e.g., shape, color, quantity, or design). In the current version based on Xie and Pisano (2019), the WCST consisted of four stimulus cards and 128 response cards that depicted various symbols (crosses, circles, triangles, or stars), in different colors (red, blue, yellow, or green) and numbers of figures displayed (one, two, three, or four). After each sorting, participants were given feedback so that they could shift to another rule if the feedback is negative. In the computerized test programmed by E-prime 2.0, each trial began with a fixation “ + “ for 1000 ms, and then a response card and four stimulus cards appeared for 3000 ms during which participants were required to sort the response card by pressing the designated keys. After each sorting, the feedback would be given for 1000 ms before the next trial. One important design is that after a few trials (5–9), the implied rule would change without informing participants.

#### The Flanker task

Flanker task (Eriksen and Eriksen, 1974) was used to measure inhibition and monitoring of cognitive control by judging the direction of the red target chevron, which is interfered with by other surrounding symbols (Festman and Münte, 2012). In the neutral condition, the target was flanked by black diamonds, which had no relevance to the target red chevron. In the congruent condition, the target was flanked by chevrons in the same direction. In the incongruent condition, the red target chevron was flanked by chevrons in the opposite direction.

The computerized task, which was designed and operated on E-Prime 2.0., had two blocks. In the practice block, there were nine trials with feedback. Participants could not enter into the formal block until they completed the practice block with accuracy above 80% to ensure full attention to the task. In the formal block, participants were required to decide on the direction of the red target chevron as accurately and fast as possible by pressing designated keys. Each trial began with a fixation of “ + “ for 250 ms. Then an experimental stimulus was randomly presented for 2000 ms or the designed keys were pressed before the next trial started again. There were altogether 108 trials with the three conditions evenly distributed in the formal experiment, without feedback for each trial.

## Results

### Demographic features

The demographic information (see Table 1) of all the participants showed that the average age was 21.33 (*SD* = 2.15), indicating that our participants were young adults whose cognitive control ability was at the best level. Paternal education was 3.55 (*SD* = 1.18), and maternal education was 3.33 (*SD* = 1.20), which reveals that their SES was at a moderate level. For language proficiency, L1 reached 8.11 (out of 10, *SD* = 0.64), and L2 was 6.21 (out of 10, *SD* = 0.88), suggesting that our participants were unbalanced Chinese–English bilinguals. Furthermore, their objective L2 proficiency test result showed that the average score was 31.37 (*SD* = 7.34).

**TABLE 1:** Demographic information of all participants.

	*N*	Minimum	Maximum	Mean	*SD*
Demographic background					
Age (years)	111	17.00	28.00	21.33	2.15
Paternal education (1–7)	111	2.00	7.00	3.55	1.18
Maternal education (1–7)	111	1.00	6.00	3.33	1.20
Language proficiency					
L1 proficiency (1–10)	111	6.00	10.00	8.11	0.64
Self-rated L2 proficiency (1–10)	111	4.00	8.00	6.21	0.88
L2 category verbal fluency	111	16.00	51.00	31.37	7.34

### Garden path effect

In order to find out whether there is a garden path effect among all the participants, we compared whether there is a difference between garden path sentence processing and normal sentence processing. For the sentence processing task, the proportion of correct comprehension of garden path sentences as well as the normal sentence was calculated as accuracy, and the response times of correct comprehension were calculated as processing speed. Higher accuracy and faster response times are indicators of better sentence processing performance. The results of these two types of sentence processing are shown in Table 2.

**TABLE 2:** Means and standard deviations for the performance of garden path sentence and normal sentence.

	Garden path sentence		Normal sentence		*t* value	*p*-value
	*Mean*	*SD*	*Mean*	*SD*		
Accuracy	36.60	13.58	79.28	12.24	–20.995	0.000
RTs	2900	565	2627	511	6.519	0.000

As shown in Table 2, the accuracy of garden path sentence processing was significantly lower than that of normal sentence processing, 36.60 (*SD* = 13.58) vs. 79.28 (*SD* = 12.24), *t* = –20.995, *p* < 0.001, which suggests that the participants encountered the so-called “garden path effect” and experienced more difficulties in processing garden path sentences. Meanwhile, we compared the response times of the correct comprehension of both types of sentences. Similarly, the result showed that the mean response times of garden path sentence processing are longer than that of normal sentence processing, 2900 ms (*SD* = 565) vs. 2627 ms (*SD* = 511), *t* = 6.519, *p* < 0.001, indicating that the so-called “garden path effect” can also be observed in terms of processing speed. In short, for the 111 Chinese–English bilinguals that included freshmen, sophomores, juniors, and seniors, the results of both accuracy and response times comparisons between the two types of sentences revealed that garden path sentences incurred more difficulties in ambiguity resolution, as reflected by lower accuracy and longer response times.

### Effect of cognitive control on garden path sentence processing

In order to examine whether different components of cognitive control affect garden path sentence processing, we divide the participants into high and low groups differing in working memory, inhibition, conflict monitoring, and mental set shifting, and then compare the performance differences in processing garden-path sentences between groups, with other variables strictly matched.

#### Working memory and garden-path sentence processing

According to the scores of working memory capacity, 111 participants were divided into two groups, with 31 students in each group (27.9% of the top and 27.9% of the bottom). Independent *t*-test analysis results showed that the two groups differed significantly in working memory, *t*(60) = –14.268, *p* < 0.001, and their demographic variables were matched between groups (Table 3).

**TABLE 3:** Comparisons between working memory groups.

	Low WM group (*n* = 31)	High WM group (*n* = 31)	*t* value	*p*-value
	Mean (*SD*)	Mean (*SD*)		
Demographic background				
Age (years)	21.61 (2.06)	21.23 (1.99)	0.751	0.455
Paternal education (1–7)	3.35 (1.25)	3.64 (0.91)	–1.042	0.302
Maternal education (1–7)	3.42(1.38)	3.42 (1.02)	0.000	1.000
Language proficiency				
L1 proficiency	8.06 (0.73)	8.29 (0.64)	–1.295	0.200
Self-rated L2 proficiency	6.06 (0.85)	6.39 (0.88)	–1.463	0.149
L2 category verbal fluency	29.84 (8.06)	31.03 (7.49)	–0.604	0.548
Working memory	7.94 (0.54)	10.74 (0.96)	–14.268	0.000
Garden path accuracy	36.89 (14.64)	35.08 (14.84)	0.484	0.630
Normal sentence accuracy	75.40 (14.15)	78.43 (13.68)	–0.855	0.396
Garden path RTs	3067 (458)	2635 (680)	2.934	0.005
Normal sentence RTs	2716 (471)	2387 (579)	2.450	0.017

As Table 3 shows, there were no group differences in the demographic and linguistic background such as age, SES, L2 proficiency, L2 proficiency, etc. (*p*s > 0.149). However, there were differences in working memory between groups. The high working memory group had greater capacity than the low working memory group (10.74 vs. 7.94), *t*(60) = –14.268, *p* < 0.001. More importantly, in order to verify whether differences in working memory would be associated with differences in sentence processing, we compared the accuracy and response times (RTs) between the two groups on both garden path sentences and normal sentences. The independent samples *t*-test analyses results showed that the high working memory group was faster in processing both garden path sentences and normal sentences (2635 ms vs. 3067 ms; 2387 ms vs. 2716 ms), with *t*(60) = 2.934, *p* = 0.005, *t*(60) = 2.450, *p* = 0.017 respectively. However, there were no group differences in accuracy on garden path sentences and normal sentences, with *p* = 0.630, and *p* = 0.396 respectively. These results suggest that working memory has a significant effect on garden path sentence processing. Higher working memory is associated with faster processing speed.

#### Inhibition and garden-path sentence processing

Of all the participants, two groups were formed based on their performance on inhibition (RT difference between incongruent and congruent trials in the Flanker task), with 31 students in each group (27.9% of the top and 27.9% of the bottom). Independent samples *t*-test analysis results showed that the two groups differed significantly in inhibition (26.46 ms vs. 88.36 ms), *t*(60) = –14.586, *p* < 0.001, and their demographic variables were matched between groups (Table 4).

**TABLE 4:** Comparisons between inhibition groups.

	Low inhibition group (*n* = 31)	High inhibition group (*n* = 31)	*t* value	*p*-value
	Mean (*SD*)	Mean (*SD*)		
Demographic background				
Age (years)	21.13 (2.06)	21.61 (2.38)	0.856	0.395
Paternal education (1–7)	3.55 (1.15)	3.55 (1.21)	0.000	1.000
Maternal education (1–7)	3.23 (1.14)	3.29 (1.13)	0.223	0.324
Language proficiency				
L1 proficiency (1-10)	8.26 (0.51)	8.06 (0.68)	–1.264	0.211
L2 proficiency (1-10)	6.29 (0.78)	6.13 (0.76)	–0.821	0.415
L2 category verbal fluency	32.06 (8.03)	31.10 (6.27)	–0.529	0.599
Inhibition	88.36 (14.60)	26.46 (18.61)	–14.568	0.000
Garden path accuracy	39.31 (14.42)	36.90 (16.00)	–0.665	0.509
Normal sentence accuracy	75.40 (14.15)	78.43 (13.68)	–0.637	0.527
Garden path RTs	2808 (494)	2832 (823)	0.137	0.891
Normal sentence RTs	2582 (511)	2570 (609)	–0.086	0.932

Similarly, accuracy and response times were compared between groups. Independent samples *t*-test analyses results showed that the high inhibition group did not perform better than the low inhibition group on the accuracy of garden path sentences processing and normal sentences processing, *t*(60) = –0.665, *p* = 0.509, *t*(60) = –0.637, *p* = 0.527 respectively. Moreover, comparisons on RTs of garden path and normal sentence processing did not show significant differences either, *t*(60) = 0.137, *p* = 0.891, *t*(60) = –0.086, *p* = 0.932 respectively. These results together showed that there were no group differences either in accuracy or RTs of processing garden path sentences and normal sentences, indicating that inhibition affects neither garden path nor normal sentence processing.

#### Conflict monitoring and garden-path sentence processing

The overall RTs in the Flanker task were taken as an indicator of conflict monitoring. Faster RTs mean a better ability for conflict monitoring. We classified the participants into low monitoring group and high monitoring group (27.9% of the top and 27.9% of the bottom, 31 participants in each group), 571 ms vs. 445 ms, *t*(60) = –13.364, *p* < 0.001, with other variables matched. Detailed information between groups is presented in Table 5.

**TABLE 5:** Comparisons between conflict monitoring groups.

	Low monitoring group (*n* = 31)	High monitoring group (*n* = 31)	*t* value	*p*-value
	Mean (*SD*)	Mean (*SD*)		
Demographic background				
Age (years)	21.00 (1.84)	20.90 (1.96)	–0.200	0.842
Paternal education (1–7)	3.32 (1.17)	3.74 (1.31)	1.328	0.189
Maternal education (1-7)	3.39(1.28)	3.48(1.21)	0.306	0.761
Language proficiency				
L1 proficiency (1–10)	8.06 (0.63)	8.03 (0.55)	–0.215	0.830
L2 proficiency (1–10)	6.20 (0.98)	5.97 (0.84)	–0.976	0.333
L2 category verbal fluency	29.74 (7.56)	32.42 (6.78)	1.468	0.147
Conflict monitoring	571 (46)	445 (26)	–13.364	0.000
Garden path accuracy	36.69 (11.50)	38.31 (13.86)	0.499	0.620
Normal sentence accuracy	79.64 (9.12)	78.63 (9.38)	–0.429	0.669
Garden path RTs	3092 (532)	2737 (601)	–2.461	0.017
Normal sentence RTs	2698 (533)	2598 (481)	–0.780	0.438

As Table 5 shows, there are no group differences in demographic features such as age, and parental education (*p*s > 0.189), and there are no group differences in language proficiency (*p*s > 0.147). In order to examine whether conflict monitoring has effect on sentence processing, accuracy and response times were compared between groups. Independent samples *t*-test analyses results showed that the high monitoring group did not perform better than the low monitoring group on the accuracy of garden path sentences processing and normal sentences processing, *t*(60) = 0.499, *p* = 0.620, *t*(60) = –0.429, *p* = 0.669 respectively. Moreover, comparisons on RTs of normal sentence processing showed no significant differences either, *t*(60) = –0.780, *p* = 0.438. However, a comparison of RTs of garden path sentences processing showed that the high monitoring group performed significantly faster than the low monitoring group (2737 ms *vs.* 3092 ms), *t*(60) = –0.2.461, *p* = 0.017. These results together suggest that monitoring of cognitive control does not affect sentence processing accuracy or normal sentence RTs, but it significantly affects RTs of garden path sentence processing.

#### Shifting and garden-path sentence processing

According to the completed categories of WCST, which measures mental set shifting ability of cognitive control, participants were categorized into the low shifting and the high shifting group (27.9% of the top and 27.9% of the bottom, with 31 participants in each group), 4.32 categories *vs.* 10.13 categories, *t*(60) = 14.257, *p* < 0.001, with other variables matched (see Table 6).

**TABLE 6:** Comparisons between shifting groups.

	Low shifting group (*n* = 31)	High shifting group (*n* = 31)	*t* value	*p*-value
	Mean (*SD*)	Mean (*SD*)		
Demographic background				
Age (years)	20.55 (1.73)	21.26 (1.95)	1.517	0.135
Paternal education (1–7)	4.00 (1.29)	3.58 (1.29)	–1.282	0.205
Maternal education (1–7)	3.71 (1.42)	3.42 (1.12)	–0.895	0.375
Language proficiency				
L1 proficiency (1-10)	8.06 (0.57)	8.00 (0.77)	–0.373	0.711
L2 proficiency (1-10)	6.13 (0.88)	6.11 (0.79)	–0.076	0.940
L2 category verbal fluency	31.94 (6.81)	32.10 (8.86)	0.080	0.936
Shifting	4.32 (1.60)	10.13 (1.61)	14.257	0.000
Garden path accuracy	39.31 (15.74)	37.10 (10.70)	0.661	0.511
Normal sentence accuracy	78.02 (14.87)	79.03 (10.65)	0.307	0.760
Garden path RTs	2813 (719)	2984 (334)	1.201	0.235
Normal sentence RTs	2663(687)	2630 (403)	–0.230	0.819

Independent samples *t*-tests were conducted to compare group differences. Results showed that the two groups did not differ in their demographic and linguistic background (*p*s > 0.135). Furthermore, comparisons between groups on sentence processing accuracy and RTs did not show differences (*p*s > 0.235). All these results showed that shifting does not have an effect on sentence processing, either on accuracy or response times, either on garden-path sentences or normal sentences.

To sum up, the overall results of data analyses showed that the garden path effect is significant: processing garden path sentences consumes longer time and incurs lower accuracy among all the participants. Moreover, only some components of cognitive control have effects on garden path sentence processing. Higher working memory and conflict monitoring are associated with faster processing speed in processing garden path sentences, whereas inhibition and shifting are not.

## Discussion

In light of the increasing attention to the role of cognitive control in garden path sentence processing, the current study was designed to verify the garden path effect relative to non-garden-path sentences and to further explore how different components of cognitive control might be related to garden path sentence processing. It is found that, firstly, the so-called “garden path effect” existed among the 111 undergraduate Chinese–English bilinguals, both in terms of accuracy and RTs; secondly, high working memory and high conflict monitoring groups performed significantly better than the low groups in the comprehension of garden path sentences. These findings are generally consistent with the theoretical hypothesis that cognitive control has a significant role in garden path sentence processing, but is reflected in differential patterns in regards to different components.

### Evidence of garden path effect

The results of the current study are in line with previous studies concerning the differences between garden path sentence and normal sentence processing, revealing clear evidence of the garden path effect in L2. Particularly, the participants in the current study were young adult Chinese–English bilinguals (*M* = 21.33), whose SES was of moderate status (*M* = 3.33–3.55), L1 as a native language, and L2 was of moderate level (*M* = 6.21 out of 10). The results that participants processed L2 garden path sentences more difficult than normal sentences are consistent with previous findings, as reflected by longer RTs (2900 vs. 2627) and lower accuracy (36.60 vs. 79.28). A few previous studies have found that participants encounter similar difficulties during the processing of L2 garden path sentences, as reflected by lower accuracy as well as longer RTs (e.g., Cheng, 1998; Gu and Cheng, 2010; Hou and Feng, 2017). For example, in the research of Gu and Cheng (2010) from China, 106 English major students participated in the English (L2) garden path sentence comprehension test adapted from Christianson et al. (2001). Results showed that the error rate of the garden path sentence was much higher than that of the normal sentence. Similar results were also found in studies on L1 garden path sentence processing (e.g., Novick et al., 2014; Teubner-Rhodes et al., 2016). In the experiment of Teubner-Rhodes et al. (2016), for example, both the healthy adult balanced Spanish–Catalan bilinguals and the Spanish-speaking monolinguals showed longer response times in comprehending L1 garden path sentences, with lower accuracy compared with normal sentences comprehension. These findings suggest that no matter for L1 or L2 garden path sentences processing, the so-called “garden path effect” coherently exists in processing sentences that require ambiguity resolution. As our participants were Chinese, combined with similar findings from earlier studies, we could conclude that this garden path effect is prevalent across different populations.

### Cognitive control and garden path sentence processing

The findings in our study clearly showed that the high working memory group performed faster than the low working memory group, although both groups achieved the same level of accuracy. This result is consistent with findings from previous studies in that the relationship between working memory and processing of garden path sentences is significant (Christianson et al., 2006; Teubner-Rhodes et al., 2016; Hussey et al., 2017; Yoo and Dickey, 2017), which verifies the positive role of working memory in resolving garden path sentences. However, the group difference in accuracy was null, which contradicts the finding that the accuracy of garden path sentences processing is significantly lower than that of normal sentences (Choi and Trueswell, 2010; Key-DeLyria and Altmann, 2016; Pozzan and Trueswell, 2016; Yoo and Dickey, 2017). Theoretically, higher working memory capacity may help parsers perform better in processing ambiguous structures by mobilizing cognitive resources to a greater extent, but the result does not show any effect on accuracy. One possibility of the null effect was that the participants from this study were all young adults between 21 and 28 years of age, and their cognitive control mechanism was at its best, so they might reach a ceiling effect when processing the garden path sentences. Future studies may incorporate variances of task difficulty to further investigate this issue.

More importantly, the current study has brought us some new findings. Working memory has been reported by previous research to play a significant role in garden path sentence processing. However, few studies have focused on the roles of other components of cognitive control. In the current study, apart from working memory, it has been confirmed that conflict monitoring also has a significant role in the garden path sentence processing. The high conflict monitoring group performed much faster than the low monitoring group in garden path sentence processing but not in normal sentence processing, suggesting a significant role of conflict monitoring in the resolution of ambiguity. Conflict monitoring has been seen as the ability to monitor whether there is conflict (or error detection) and whether there is a need to utilize cognitive resources to resolve conflict resolution (Costa et al., 2009). In resolving ambiguity in the garden path sentences, participants need to monitor whether there is a conflict of reasonable interpretation as the sentence unfolds and if so whether to adopt cognitive control system to override a more dominant response or flexibly switch to another possible interpretation. It is likely that participants with a higher level of conflict monitoring are more able and more efficient to monitor or detect conflict from the context. This advantage may lead to better performance in the processing of garden path sentences, which is reflected by faster processing speed in the current study. Our study is the first of its kind to verify the role of conflict monitoring in processing garden path sentences. Quite a few studies have found that bilinguals (or higher level of L2 proficiency) are more advantageous than monolinguals in conflict monitoring as they always need to monitor when to use the target language and when to resolve the conflict if the non-target language is interfering (Hilchey and Klein, 2011; Xie and Pisano, 2019). These findings suggest that conflict monitoring and language processing are closely interwoven, particularly when there is a need for conflict resolution (such as in processing garden path sentences).

However, our study did not find significant roles of inhibition and shifting in processing garden path sentences through young-adult group comparisons, which is not similar to the findings of Engelhardt et al. (2017). In their study, they found that inhibition played a marginally significant role in garden path sentence processing through within-group factor analysis. Individuals (young adults) with poorer inhibitory control were less likely to answer the comprehension questions correctly. However, they interpreted that ambiguity resolution does not heavily rely on inhibition as most inhibition is shared with individual differences in intelligence, and it is also possible that the interpretations of syntactic ambiguity are parallel (e.g., MacDonald et al., 1994; Hsieh and Boland, 2015) so there is no need to inhibit. However, the null effect of inhibition is consistent with prior research (e.g., Christianson et al., 2006; Engelhardt et al., 2008).

The role of shifting has not been much examined in previous literature. Our study did not observe the effect of shifting ability on processing garden path sentences, which is similar to the findings from Engelhardt et al. (2017). However, Woodard et al. (2016) found that two measures of cognitive flexibility/shifting were related to their ambiguity resolution abilities, i.e., switching in the Card Sorting test and switching cost in the Flanker. We speculate that this inconsistency may be related to task difficulty variations, both in ambiguity resolution tasks and non-linguistic shifting tasks. For example, if the time limit is not so demanding (e.g., an easy task but a long time limit for response) for switching/opting to a more possible interpretation in the ambiguity resolution process, the group differences may not emerge. In our experimental design, the garden path sentences may be easy for our participants and the time limit (5000 ms) may not be so strict. More future studies are therefore encouraged to verify how this component of cognitive control may impact ambiguity resolution in different linguistic and non-linguistic contexts. Moreover, since our participants were Chinese students who were learning English as a foreign language, it is also worthwhile to further verify the question of whether the same results would be obtained with ESL learners from a language background other than Chinese.

To wrap up our discussion, our study has expanded the role of cognitive control in garden path sentence processing to its diversified components, i.e., working memory, conflict monitoring, inhibition, and shifting, and proved that working memory and conflict monitoring significantly affect garden path sentences processing. Although our design is quasi-experimental, however, some studies have shown that it is possible to increase individuals’ ability of language processing by training their cognitive control, further clarifying the cause and effect relation between cognitive control and language processing. For example, in Hussey et al. (2017), individuals were trained on different versions of the N-back task. After the training intervention, they found that high conflict training produced benefits on memory and language measures, which indicates a causal role of domain-general cognitive control in linguistic and non-linguistic performance. Overall, these limited studies on the relationship between cognitive control and garden-path sentence processing provide us with a new perspective to probe into the nature of language and language learning and call for longitudinal studies in future research. Another point to be noted, which is a limitation of our study and calls for improvement in future study, is that our study did not use a more objective measure of proficiency as LexTale English (Lemhöfer and Broersma, 2012), in addition to the Semantic Verbal Fluency Task. The participants in our study, who had been learning English (L2) for more than 10 years, only reported self-rated L1 and L2 proficiency and completed L2 Semantic Verbal Fluency Task. We believe that more objective test of language proficiency (both L1 and L2) would have added more strength to our findings.

## Conclusion

In this research, we adopted the garden path sentence comprehension task and multiple cognitive control tasks, i.e., Digit Span task, WCST, and Flanker task, to verify the garden path effect and examine the roles of different components of cognitive control in garden path sentences processing. The main findings are presented in the following aspects. Firstly, the Chinese English learners encountered difficulties in the processing of garden path sentences. The garden path effect was observed. Secondly, among several components of cognitive control, high working memory and conflict monitoring groups significantly outperformed the counterpart groups in garden path sentence processing, which indicates that working memory and conflict monitoring have significant impacts on garden path sentence processing. Thirdly, however, no effect of inhibition and shifting was found in our study. Longitudinal studies are encouraged to further explore why and how different components of cognitive control may affect garden path sentence comprehension under different linguistic contexts.

## Data availability statement

The raw data supporting the conclusions of this article will be made available by the authors, without undue reservation.

## Ethics statement

The studies involving human participants were reviewed and approved by the academic committee of Jiangxi Normal University. The patients/participants provided their written informed consent to participate in this study.

## Author contributions

ZX designed the whole research and finalized the manuscript. GZ drafted and revised the manuscript, SZ drafted the manuscript, and JW conducted the experiment and collected the data. All authors contributed to the article and approved the submitted version.
